# Informed Consent and Shared Decision-Making: A Requirement to Disclose to Patients Off-Label Prescriptions

**DOI:** 10.1371/journal.pmed.0050223

**Published:** 2008-11-11

**Authors:** Michael Wilkes, Margaret Johns

## Abstract

Michael Wilkes and Margaret Johns argue that the doctrine of informed consent should require doctors to disclose to patients when they are prescribing a drug off-label.

## Introduction

A 9-year-old with cerebral palsy received an injection of the neurotoxin “Botox” to relieve muscle spasms. This off-label use was legal but not approved by the US Food and Drug Administration (FDA) for this indication. People with headaches have also received Botox injections as a legal, but unapproved, treatment—in this case the FDA is investigating whether the manufacturer actually promoted the drug for this indication. In fact, the drug has some significant dangers leading to hospitalizations and deaths [1].

A more familiar instance of off-label drug use would be the example of a 47-year-old male presenting to his doctor with lower back pain. The doctor, having previously suggested over-the-counter medications, prescribes a drug to ease the pain. The doctor tells the patient to take the drug three times a day, but provides no other information. In this case, a reasonable person might wish to be told: (1) that the prescribed drug gabapentin was approved by the FDA only to treat seizures in epilepsy—not for back pain; and (2) that no reliable research supports using the drug for back pain.

These examples are not uncommon, yet current practice does not require or even suggest that doctors disclose any of these facts to their patients. This article argues that as an extension of the legal doctrine of informed consent and the ethical duty of shared decision-making (SDM), patients should be told when a drug is being prescribed “off-label;” that is, it has not been approved for the indication and is being used experimentally.

**Summary Points**

- Off-label prescriptions are those that do not comply with the FDA-approved use for the drug. While they are legal and account for roughly half of all prescriptions written today, often they are not supported by sound scientific evidence.
- In addition, they have the potential to drive up the cost of health care and expose patients to unnecessary risks and uncertain outcomes. Legal and ethical principles require physicians to inform patients about risks of medical treatments.
- We propose that the doctrine of informed consent be rigorously applied to require doctors to disclose to patients when they are prescribing a drug off-label.
- Providing full disclosure to patients and encouraging them to share in decision-making in situations of medical uncertainty is vital to respecting their autonomy.

## Off-Label Prescribing

Because a basic premise of the US Federal Food, Drug, and Cosmetic Act is that manufacturers are prohibited from marketing drugs or devices without FDA approval, the public commonly assumes that all uses of prescription drugs have been approved by the FDA. However, after a drug is approved for one set of indications, researchers and doctors often discover new applications for it. Even when the FDA approves a drug for a single, specific use, doctors may legally prescribe the drug to *any* patient for *any* use. Physicians are not restricted to prescriptions that comply with the FDA approval. The FDA considers such treatments “off-label” because substantial evidence regarding their safety and efficacy has not been presented or evaluated. But such uses are perfectly legal. In fact, FDA policy explicitly states that “once a [pharmaceutical] product has been approved for marketing, a physician may prescribe it for uses in treatment regimes of patient populations that are not included in the approved labeling” [2]. Indeed, as the Supreme Court has recognized, off-label prescribing “is an accepted and necessary corollary of the FDA's mission to regulate in this area without directly interfering with the practice of medicine” [3].

There are many examples of responsible, off-label prescribing. Specifically, pediatric prescriptions are frequently off-label because many drugs have not been tested on children. Aspirin was widely prescribed to reduce the risk of heart attack long before it was FDA-approved for this purpose. Off-label uses are widespread in oncology, and off-label, antiretroviral combination therapies have saved many AIDS patients. In short, off-label drug prescribing is a significant part of mainstream medicine [4].

While off-label prescriptions are common and sometimes necessary, they also present significant risks. Often, the drug has not been proven safe or effective for treating the patient's condition [5,6], and off-label prescribing usually “occurs without scientific support” [6]. FDA panels have found that off-label uses can be dangerous [7,8]. For example, doctors wrote 18 million prescriptions for the off-label use of fenfluramine for weight loss before it was discovered that thousands of people suffered heart valve damage from it [7].

Off-label prescribing has potential consequences for both the individual patient and for our health care system. First, an off-label prescription may be ineffective or downright detrimental in treating the medical condition. By definition, no governmental body has evaluated the effectiveness or safety for the off-label indication, and often there is no rigorous evidence base to properly evaluate the drug. Second, accepting poorly studied therapies heightens the risk of overmedication and drug interactions. And finally, the drugs prescribed off-label are often more expensive than an off-patent or generic medicine.

Furthermore, there is the potential for an escalating financial burden on our health care system. According to a report by the National Association of Attorneys General, the single biggest factor driving the increase in health care costs is the price of prescription drugs [9,10]. It is likely that a significant part of prescription drug cost is due to increased off-label prescribing of on-patent drugs [11,12]. Prescription drugs are the fastest growing part of our health care costs [9,10], with spending increasing at double-digit rates annually from 1997 to 2005 [9]. Between 1990 and 2002, the amount spent on prescription drugs in the United States increased 4-fold from US$40.6 billion to US$162 billion [9].

In practice, the FDA exercises little oversight on off-label promotion. Making matters worse, pharmaceutical companies often publish questionable research using medical education and communication companies (MECCs) [10]. These MECCs conduct flimsy research and present continuing medical education courses on off-label uses [10]. Often research is written by company-paid ghost writers but bears the name of a medical school faculty member paid generously for the use of their name [13,14]. These “articles” are then presented to doctors at free “educational” programs [10,15]. This strategy is often used to promote off-label, on-patent uses. Not surprisingly, these studies are heavily biased in favor of the company's product and aggressively disseminated to practicing physicians using the army of pharmaceutical sales representatives. Unfortunately, lax regulation has sometimes led to illegal over-promotion of off-label therapies.

## Informed Consent, Shared Decision-Making, and Off-Label Prescriptions

Given that off-label prescribing of drugs may expose patients to unnecessary risks and may result in the prescribing of expensive new drugs when older ones are equally effective, cheaper, and safer, it is reasonable to apply the ethical mandate for SDM to doctors and require that health care providers disclose off-label prescribing to patients and seek their consent to the off-label use. In the US there are two grounds for requiring these discussions, one legal (informed consent) and one ethical (SDM).

### A. The legal doctrine of informed consent.

While laws and policies in other countries may differ, in the US today, legal standards require physicians to obtain informed consent from a person before performing a test or starting a treatment—particularly a treatment that involves some uncertainty [16]. The doctrine of informed consent reflects the value we place on patient autonomy. Until the early twentieth century, doctors were not required to inform their patients of the risks and alternatives to a proposed treatment. The assumption was that doctors knew what was best for patients and that patients were sufficiently protected by their doctors' interest in their well-being. Over time, as patients asserted greater autonomy rights, the law evolved to impose a duty on doctors to make “those disclosures which a reasonable medical practitioner would make under the same or similar circumstances” [17]. Some later cases took patient autonomy a step further by refocusing the analysis on the information the patient would want to know rather than on the information a doctor would customarily disclose [18]. According to the doctrine of informed consent, the doctor is required to disclose the nature of an intervention, pros and cons of intervention, alternatives to intervention, and pros and cons of alternatives. This is where the informed-consent doctrine stands today: some states use the reasonable-doctor standard while others use the reasonable-patient standard. Under either approach, the law recognizes a “therapeutic privilege” when full disclosure would be detrimental to a patient's total care and best interests.

Given that patients assume a drug prescribed by their doctor (1) has been proven safe and effective, (2) is FDA approved, and (3) is supported by scientific evidence [6], the question becomes: should an off-label therapy be disclosed to satisfy the requirements of informed consent? Would this information likely affect a patient's decision to take the medicine [19]? In other words, are these risks that a reasonable person might wish to know about before accepting a prescription? If so, then requiring disclosure makes sense. It is important to note that FDA approval is not a panacea, nor does FDA approval guarantee safety and effectiveness (e.g., Vioxx and ezetimibe are just two recent examples of drugs approved by the FDA that turned out to pose dangers to users). Despite these problems, FDA approval is one step better than no approval. Of course, disclosure would absolutely be required if the patient was involved in a formal drug research trial. In many ways the patient given an off-label prescription is involved in an “*n* of one” research trial and should be required to provide informed consent.

To date, no court has required a doctor to disclose that a therapy is off-label. In the few cases considering the issue, the courts have concluded that FDA classifications “do not speak directly to the medical issues” [20]. Those who oppose a disclosure requirement argue that disclosure would unduly frighten patients who would then refuse optimal treatments [21,22]. They have also claimed that requiring disclosure would unduly burden doctors whose attention would be diverted away from patient care, as they would be forced to read government materials to determine the risks, benefits, and approval status of each drug [9].

These concerns are minor and theoretical compared to the real imperative of patient self-determination. Does concern about frightening patients preclude discussion about surgery or other medical treatments? The notion that patients cannot make competent health care decisions when provided truthful information flies in the face of the values supporting the doctrine of informed consent. Further, in the exceptional case where disclosure would be detrimental to the patient's health, the therapeutic privilege already allows a doctor to withhold the information. Rather than routinely withholding this information from competent patients, doctors should be required to routinely disclose it to promote patient autonomy, ensure informed consent, and engage in SDM.

Moreover, determining approval status is hardly an undue burden. It is a simple task to determine the FDA status of a drug and approved indications. The information is readily available in the approved product label, the Physicians' Desk Reference, and on-line services. Since 2006, the FDA has required drug manufacturers to provide the FDA-approved uses in a computer format that is readily accessible to doctors' computers and hand-held devices, and this information is a part of some electronic medical records systems [23]. In addition, the FDA is standardizing and simplifying the approval information to make it even more readily available and understandable [9]. Increasingly expert medical opinion supports the feasibility of disclosing off-label uses. Specifically, in 2006, a multidisciplinary group developed a policy for off-label prescribing for medical centers [24]. It concluded that for off-label uses—where the prescribing is not sufficiently tested to allay concerns about safety, efficacy, and cost-effectiveness—“physicians . . . must meet their ethical obligations by ensuring that the patient is informed and provides consent prior to administering the drug” [9,16,25]. One insurance company already provides a form for physicians to use in obtaining informed consent for off-label uses [26]. So, it appears that off-label disclosure is practically feasible.

### B. The ethical requirement of SDM.

The ethical requirement for SDM goes even further than the legal doctrine of informed consent. Initially, SDM involves a discussion to determine a patient's desire to participate in decision-making. It then involves a presentation of information about reasonable options in terms patients can understand. Finally, it involves *both* the doctor and the patient arriving at a mutually acceptable decision based on their shared knowledge and values. Characteristics of decisions that lend themselves best to SDM are outlined in Box 1.

**Box 1. Characteristics of Decisions that Lend Themselves Best to Shared Decision-Making**

- Decisions where the effectiveness of the outcome is uncertain;
- Decisions where the risks and benefits are sizeable or nearly equal;
- Decisions where the patient is able and willing to participate; and
- Decisions where the patient can understand the trade-offs between different approaches.

Off-label prescribing seems the poster child for SDM, where some indications suggest a benefit but others suggest known and unknown risks—in other words, medical uncertainty. Faced with medical uncertainty, doctors owe patients the ethical duty to inform them of the uncertainty and offer them choices. It's one thing to prescribe a drug off-label for a serious condition when there are no other FDA-approved therapies, especially when reliable research supports the prescription. It is quite another to prescribe a drug off-label when there are safe and effective FDA-approved alternatives or when the patient's condition is not sufficiently serious to warrant the risks of an unproven and potentially dangerous treatment. However, from an ethical perspective, both cases require open, honest discussions where doctors tell their patients that the use of the drug will be off-label and thus not approved for this indication, explain the risks, potential benefits, and alternatives, and then ask patients for their permission to proceed.

At a minimum, physicians should be required to include the items in Box 2 in discussions and document that they have engaged patients in a SDM process.

**Box 2. Items to Discuss in a Shared Decision-Making Process about Prescribing**

- Whether the drug is FDA-approved for treatment of this condition or is off-label;
- Whether there is an FDA-approved alternative, including generic medication;
- Whether the off-label treatment has advantages compared to FDA-approved alternatives; and
- Whether credible research supports the off-label use.

## Conclusion

Patients need information about off-label uses to make well-informed health care decisions. The legal doctrine of informed consent should be expanded to require disclosure of off-label prescribing where the drug has not been proven safe and effective for the condition, especially where scientific evidence is inadequate and risks are substantial or unknown. The ethical requirement of SDM should be expanded to require discussions of off-label uses under the same circumstances. Requiring disclosure will protect patient autonomy and educate patients about alternatives and risks, leading to improved health care decisions.

## Abbreviations

FDA: Food and Drug Administration
SDM: shared decision-making

## References

[pmed-0050223-b001] Begley S (2008 April 21). A new reason to frown. Newsweek. http://www.newsweek.com/id/131749?tid=relatedcl.

[pmed-0050223-b002] Arbitlit DC, Fleishman W (2005). The risky business of off-label use. Trial.

[pmed-0050223-b003] US Supreme Court (2001). Buckman Co. v. Plaintiffs' Legal Comm. http://www.law.cornell.edu/supct/html/98-1768.ZO.html.

[pmed-0050223-b004] Steinbrook R (2006). The price of sight—Ranibizumab, bevacizumab, and the treatment of macular degeneration. N Engl J Med.

[pmed-0050223-b005] O'Reilly J, Dalal A (2003). Off-label or out of bounds? Prescriber and marketer liability for unapproved uses of FDA-approved drugs. Ann Health Law.

[pmed-0050223-b006] Radley DC, Finkelstein SN, Stafford RS (2006). Off-label prescribing among office-based physicians. Arch Intern Med.

[pmed-0050223-b007] Salbu SR (1999). Off-label use, prescription, and marketing of FDA-approved drugs: An assessment of legislative and regulatory policy. Fla Law Rev.

[pmed-0050223-b008] Stoffelmayr KJ (1996). Products liability and “off-label” uses of prescription drugs. Univ Chic Law Rev.

[pmed-0050223-b009] National Association of Attorneys General (2005). Addressing the cost and benefits of prescription drugs. 2005.

[pmed-0050223-b010] Angell M (2006). The truth about the drug companies.

[pmed-0050223-b011] Moyer CA (2005). Off-label use and the Medical Negligence Standard under Minnesota law. William Mitchell Law Rev.

[pmed-0050223-b012] Adams C, Young A (2003 November 3). Drug-makers' promotions boost off-label use by doctors. Knight Ridder Washington Bureau. http://www.accessmylibrary.com/coms2/summary_0286-8989915_ITM.

[pmed-0050223-b013] Renni D, Flanagin A (1994). Authorship! Authorship! Guests, ghosts, grafters, and the two-sided coin. JAMA.

[pmed-0050223-b014] Flanagin A, Carey LA, Fontanarosa PB, Phillips SG, Pace BP (1998). Prevalence of articles with honorary authors and guest authors in peer-reviewed medical journals. JAMA.

[pmed-0050223-b015] Kassirer J (2005). On the take: How medicine's complicity with big business can endanger your health.

[pmed-0050223-b016] Keltz JM (2003). Off-label use of prescription medication: Nursing implications. Nephrol Nurs J.

[pmed-0050223-b017] Supreme Court of Kansas (1960). Natanson v. Kline.

[pmed-0050223-b018] D. C. Circuit (1972). Canterbury v. Spence. http://www.stanford.edu/group/psylawseminar/Canterbury.htm.

[pmed-0050223-b019] Supreme Court of Oklahoma (1980). Scott v. Bradford. 606 P.2d 554. http://www.oscn.net/applications/oscn/DeliverDocument.asp?CiteID=48164.

[pmed-0050223-b020] Pennsylvania Supreme Court (2001). Southard v. Temple University Hospital.

[pmed-0050223-b021] Morris GH (2002). Discussing disclosure: Just what the doctor ordered. Ariz Law Rev.

[pmed-0050223-b022] Beck JM, Azari ED (1998). FDA, off-label drug use, and informed consent: Debunking myths and misconceptions. Food Drug Cosmet Law J.

[pmed-0050223-b023] (2006 April 20). Prescription facts to be computerized. San Francisco Chronicle.

[pmed-0050223-b024] Ansani N, Sirio C, Smitherman T, Fedutes-Henderson B, Skledar S (2006). Designing a strategy to promote safe, innovative off-label use of medications. Am J Med Qual.

[pmed-0050223-b025] Torres A (1994). The use of Food and Drug Administration-Approved medications for unlabeled (off-label) uses. Arch Dermatol.

[pmed-0050223-b026] Ophthalmic Mutual Insurance Company (2004). Informed consent for off-label use of a drug or device. http://omic.com/resources/risk_man/forms.cfm.

